# Case of Acute Hepatitis

**Published:** 1878-04

**Authors:** W. T. Belfield


### EXAMINER.

VOL. XXXVI.—APRIL, 1878.—No. 4.

### Pathological Transactions of the Chicago Medical Society.

EDITED BY DR. I. N. DANFORTH, ASSISTED BY DR. W. T. BELFIELD.

### I.

### CASE OF ACUTE HEPATITIS.

BY DR. BELFIELD.

Mrs. P——, 40 years of age, was admitted to the hospital December 20, 1877. She presented no symptoms of local lesion, but appeared to suffer from a general failure of the vital powers which had existed since her last confinement, six weeks previously. She was quite feeble and emaciated; her functions were sluggishly performed, her appetite and sleep were very poor.

No active manifestations of disease occurred until February 6th, when a chill, followed by vomiting, a dull pain in the right hypochondrium, and a high febrile movement announced the advent of an acute affection. Within 48 hours there appeared the usual accompaniments of that adynamic state known as the

"typhoid condition," viz: a dry, brown tongue; a rapid, compressible pulse; a general tremor of the muscles; sordes upon the teeth; great mental prostration, and a dark flush upon the face. Feb. 7th—There was considerable tenderness and tympanites over the whole abdomen. Feb. 8th—Jaundice was observed; the stools were found to be very light in color, while the urine was heavily laden with biliary coloring matter.

Notwithstanding the free use of stimulants the patient rapidly failed; the symptoms especially the jaundice, were much aggravated, and death occurred Feb. 17.

On section the liver was found to be smooth and somewhat enlarged in all its dimensions. From its cut surface there oozed considerable thin, watery blood. There were visible on the external as well as on the cut surfaces of the organ, numerous discolored, yellowish, soft spots varying in size from a pin's head to a pea, many of which, as was evident to the unassisted eye, were perforated by the interlobular veins and veinlets.

Microscopic examination revealed around the interlobular vessels an accumulation of young, indifferent cells, which filled completely the interlobular spaces and, indeed, encroached largely upon the acini themselves; the vessels, also, seemed somewhat compressed. There appeared to be no intercellular substance, except that occasional fibers were observed to traverse the masses of cells in the immediate vicinity of the vessels.

The liver-cells themselves were much swollen, rounded and quite cloudy from the presence of granular matter. Many of the nuclei were obscured by this cloud, while other cells presented each two distinct nuclei and nucleoli. Occasional free nuclei were also observed. The liver cells appeared to be separated from one another by considerable spaces, which contained, however, no other structure than granules.

Differentiation between the proper cells of the lobules and the adventitious cells of the interlobular growth, was rendered easy by the fact that the former were deeply tinged with biliary matter, while the latter were quite destitute of color.

